# Cold vs. Heat

**Published:** 1860-12

**Authors:** 


*Cold versus Heat.*—The annual deaths by cold and by burns in this country follow a curious law of progression when their frequency is compared with the temperature of the year. Thus the temperature of 1855 was low, and in that year deaths by cold amounted to 195, and deaths by burns and scalds to 3177; and in the year 1857, the temperature being high, the deaths by cold did not exceed 45, and by burns 2717. In the four years out of nine when the annual deaths by cold exceeded 100, the deaths by burns and scalds were 2826 on an average; in the four years when the annual deaths by cold were less than 100, the deaths by burns and scalds were 2710 on an average. The additional fires in cold weather, and the disposition to approach them without due caution, sufficiently explain this, while they also indicate the importance of applying widely the principle of rendering dress non-inflammable.

